# From supine to standing: in vivo segregation of myogenic and baroreceptor vasoconstriction in humans

**DOI:** 10.14814/phy2.13053

**Published:** 2016-12-30

**Authors:** Bruno Estañol, Ana Leonor Rivera, Raúl Martínez Memije, Ruben Fossion, Fermín Gómez, Katherine Bernal, Sofía Murúa Beltrán, Guillermo Delgado‐García, Alejandro Frank

**Affiliations:** ^1^ Laboratorio de Neurofisiología ClínicaDepartamento de Neurología y PsiquiatríaInstituto Nacional de Ciencias Médicas y Nutrición Salvador ZubiránMéxico CityMéxico; ^2^ Centro de Ciencias de la ComplejidadUniversidad Nacional Autónoma de MéxicoMéxico CityMéxico; ^3^ Instituto de Ciencias NuclearesUniversidad Nacional Autónoma de MéxicoMéxico CityMéxico; ^4^ Departamento de Instrumentación ElectromecánicaInstituto Nacional de CardiologíaMéxico CityMéxico; ^5^ Departamento de Medicina InternaHospital Universitario “Dr. José Eleuterio González”Universidad Autónoma de Nuevo LeónMonterreyMéxico; ^6^ Colegio NacionalMéxico CityMéxico

**Keywords:** Baroreflex vasoconstriction, baroreflex vasoconstriction, blood flow autoregulation, blood pressure autoregulation, myogenic vasoconstriction, segregation of myogenic

## Abstract

Myogenic vascular response is a form of systemic and regional vasoconstriction produced increasing the intra‐arterial pressure by gravity. Here, the vasoconstriction due to the myogenic response, induced by the gravitational action in a dependent limb, is separated from that caused by the baroreceptor reflex. Regional changes of skin blood flow (SBF), total blood volume of the finger (TBVF), pulse pressure (PP), heart rate (HR), systolic, and diastolic blood pressure (BP) were analyzed in 10 healthy young subjects in supine and upright positions. By lowering the arm in supine position, SBF decreased compared to its basal measurement, PR increased, and PP contracted, indicating arterial vasoconstriction that rise BP. TBVF increased, demonstrating an increment in venous volume. HR did not change, reflecting no action of the baroreceptor reflex. In upright position with lowered arm, there was an additional increase in BP variables, demonstrating vasoconstriction. Moreover, BP and HR showed oscillations at 0.1 Hz reflecting the entrance of the baroreceptor reflex. The action of gravity in a dependent limb in supine position induces a regional vasoconstriction and an increase of BP due to activation of the myogenic response, while the baroreceptor reflex or other neural factors do not appear to operate. In the upright position with the arm dependent, there is a further increase in regional vasoconstriction and BP with reciprocal changes in HR, indicating the entrance of the baroreceptor superimposed to the myogenic response. This study demonstrates that the myogenic and baroreceptor vasoconstriction can be separated in vivo.

## Introduction

It is widely accepted that when a normal subject assumes the upright posture there is a redistribution of the blood volume; from 600 to 800 mL are displaced to the abdomen and the lower extremities due to the action of gravity. As a consequence of the volume displacement, there is an abrupt fall in the blood pressure (BP) of about 40 mmHg (5.33 kPa) that is rapidly compensated, in about 20 to 25 sec, by the vasoconstriction of the arteries below the heart. Concomitantly, there is an increase in the heart rate (HR) of about 15 beats per minute, which is induced by the withdrawal of the vagal action and subsequently maintained by sympathetic discharges to the sinus node, thus increasing the cardiac output. There is a simultaneous increase from 80 to 100 mmHg (10.66–13.33 kPa) in venous pressure in the veins below the heart. This change is directly proportional to the distance from the veins of the lower limbs and the abdomen to the heart, specifically to the right atrium. The venous return to the right side of the heart is aided by the changes induced by the negative intrathoracic pressure (atrial suction), produced by inspiration and by the muscle contraction of the abdomen and muscles of the lower extremities (Wieling and van Lieshout 2008).

The vasoconstriction of the arteries has been attributed to an activation of the baroreceptor reflex (BRR) that induces, as a consequence of the fall of BP at the carotid sinus and aortic receptors, a sympathetic discharge to the sinus node (cardiosympathetic BRR) and to the arterial and arteriolar vessels by the BRR (vasosympathetic reflex) (Rowell 1993; Wieling and van Lieshout 2008; Aalkjær et al. 2011; Van den Munckhof et al. 2012). The compensatory increase in BP is associated with oscillations around 0.1 Hz, which are considered to be the natural oscillations of the BRR known as Mayer waves (Wieling and van Lieshout 2008). The sympathetic discharges can be recorded in the nerves that innervate the arteries and arterioles when the subject is standing (Charkodoudian and Wallin 2008). There is also the possibility that the immediate fall of BP is due to the distension of the arteries attributed to the action of the gravity on the column of blood (Kane and Sternheim 1988; Rowell 1993).

However, this classical model does not take into consideration the increase in BP induced by the pressure of gravity on the walls of the arteries and arterioles (Kane and Sternheim 1988; Richardson and Shepherd 1989; Rowell 1993; Holtz 1996). Most of the studies settle on the fact that the venous system is more distensible than the arterial system (Berne 1981; Berne and Levy 2001). However, it is also known, since the classical work of Bayliss (1902), that the arteries contract when their intraluminal pressure increases. Bayliss termed this type of vasoconstriction a “myogenic” response, meaning that the response is independent of the sympathetic nervous vasoconstriction. A large body of research has studied the myogenic response in isolated segments of arteries and arterioles, but it has been difficult to demonstrate the myogenic response in vivo in experiments in animals (McIlveen et al. 2011), and in human subjects (Davis and Hill 1999). Hence, the myogenic response in humans has not been considered as one of the fundamental mechanisms for maintaining BP during the upright posture. The purpose of the present work is to demonstrate that the myogenic response can be studied in vivo in humans and can be dissociated or segregated from the vasoconstriction induced by the BRR.

## Materials and Methods

### Subjects

The study population consisted of 10 young control subjects, 25 ± 5 years old (from 20 to 35 years old). Subjects underwent a complete physical examination by a medical doctor. They were healthy control subjects without a history of hypertension, diabetes mellitus, cardiovascular or peripheral vascular disease, peripheral, or central neurological disorders. They did not take antihypertensive, antidiabetic, sympathetic, anticholinergic, or adrenergic medications. They all had blood glucose levels below 92 mg/dL and normal muscle stretch reflexes.

### Ethical considerations

All the subjects provided written informed consent after the procedure was explained to them, emphasizing its noninvasive nature. The study was performed according to the Helsinki and Tokyo recommendations for human investigations and was approved by our institutional review board.

### Measurements

The room temperature was kept at 23^°^C. The skin temperature on the arm was kept between 34 to 36^°^C measured with an infrared thermometer (model NUB88380; range −50°C to 380°C, Nubee^®^).

Pulsatile arteriolar skin blood flow (SBF) and nonpulsatile total blood volume of the index finger (TBVF) were recorded with a red‐infrared photoplethysmograph. Red‐infrared photopletismography has been used in several studies for a long time (Burton 1939). Pulsatile and nonpulsatile SBF were measured in nondimensional units. The photopletysmograph sensed between 640 and 960 nm with a sensitivity of 20 nm. The A/D unit had a resolution of 16 bits and a sampling rate of 200 Hz. The nonpulsatile TBVF was obtained by a low‐pass filtering of the high‐frequency signal component, and allowing only the slow DC signals. The device was designed and constructed at the Department of Biomechanical Instrumentation of the National Institute of Cardiology by one of the authors (RMM) and has been used in several previous papers (Estañol et al. 2004, 2008).

Basal BP of each subject was assessed by routine sphygmomanometry after a 5‐minute rest both in a supine and in an upright position (Welch Allyn^®^ sphygmomanometer model CE0297, México City, Mexico).

Noninvasive short‐term measurements of BP were taken at each heartbeat using a Portapres^®^ device of Finapres Medical Systems (Amsterdam, Netherlands) to monitor multiple hemodynamic parameters, including systolic blood pressure (SBP), diastolic blood pressure (DBP), mean average blood pressure (MBP), pulse pressure (PP), heart rate (HR), interbeat interval (IBI), and total peripheral resistance (TPR). The signals were obtained with the probe positioned on the middle finger. The height sensor was disconnected from the device and the distance from the hand to the heart was measured manually with a measuring tape.

### Theoretical considerations

In humans, in the supine position, BP is the same at feet, head, and heart levels (Kane and Sternheim 1988; Rowell 1993). When a bipedal animal, such as a human, assumes an upright position, BP in the arteries below the heart level is increased due to the action of gravity according to the following formula:

(1) ΔBP=ρgh

where *ρ* is the density of the blood, *g* is the acceleration of the Earth's gravitational field (at sea level a constant of 9.81 m/s^2^), and *h* is the distance from the heart (Kane and Sternheim 1988; Rowell 1993; Charkodoudian and Wallin 2008). Using this equation, it is found that a change of 1 cm in *h* is equivalent to a ΔBP of 0.7 mmHg, and a distance of 50 cm below the heart equals a ΔBP of 35 mmHg. Both in supine and upright positions, the SBP at heart level is 120 mmHg in normal subjects, and can be considered as the reference basal value. The distance from the heart to the feet for humans varies normally between 1.3 to 1.5 m (Kane and Sternheim 1988). Therefore, in an upright position, the SBP of a normal subject, in the ankle stays between 200 and 220 mmHg (Kane and Sternheim 1988). If BP equals the cardiac output times the TPR, and assuming that the cardiac output does not change significantly when only the peripheral resistance is increased in a single dependent limb in the supine position, then the changes in BP are a function of the peripheral resistance of the lowered limb. In contrast, for a standing subject, a rise in the BP will also be due to an increase in the cardiac output secondary to the baroreflex‐induced tachycardia.

### Maneuvers

**Figure 1:**
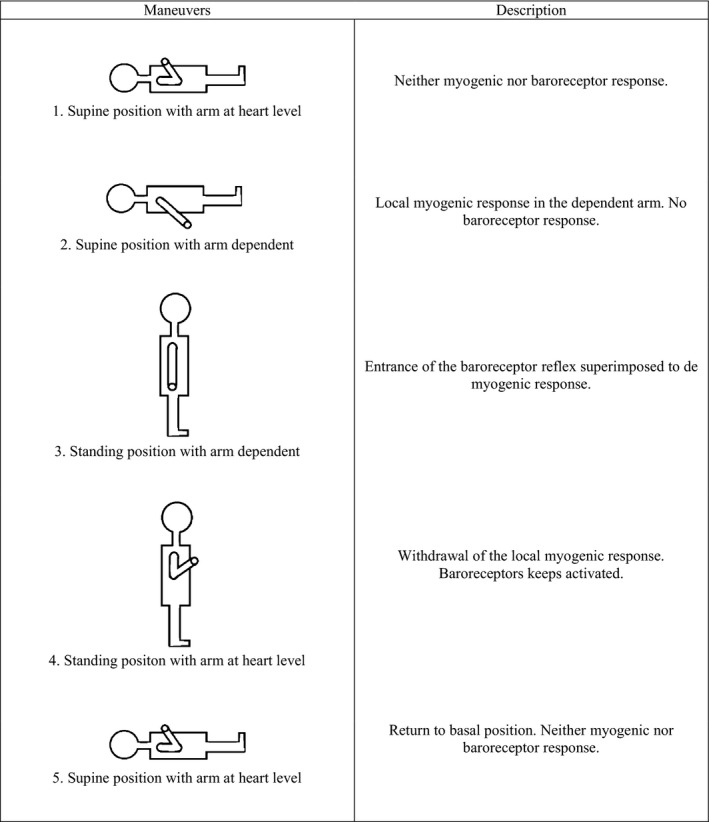
Simplified diagram of the five maneuvers and its physiological interpretation.

Subjects performed five maneuvers, each lasting 3 min. Throughout the study, the subjects were asked to remain relaxed, to move as little as possible and to breath normally. These maneuvers and their physiological meaning are depicted schematically in Figure 1. The sequence of maneuvers was as follows:

1. Supine position with the hand at heart level.
2. Supine position with one arm dependent. One arm was passively lowered by the researcher 45^°^ (50 ± 6 cm) below the heart level and supported by a lever. Subjects were asked to make no muscle effort with the dependent arm.
3. Standing position with one arm dependent. Subjects were asked to stand up as rapidly as possible, keeping the arm in the dependent position and with the same inclination as in the supine position.
4. Standing position with the hand at heart level. Subjects were asked to return the previously dependent limb to the heart level.
5. Supine position with the hand at heart level (return to the initial position).

### Mathematical analysis

For each maneuver, the statistical moments of the dynamics of all variables were calculated: mean (μ), standard deviation (SD), skewness (*sk*), and the kurtosis (κ) (Rivera et al. 2016a).

Spectral analysis was performed by taking the Fourier transform of IBI and SBP. Areas of specific frequency bands of the amplitude of the transform were determined in the low‐frequency (LF) region from 0.04 to 0.15 Hz, and in the high‐frequency (HF) region from 0.15 to 0.4 Hz. We calculated the slope of the power spectral density (PSD) by using a linear minimum squares approximation and the associated parameters LF/HF, $r_{F}=\sqrt{{\text{LF}}^{2}+{\text{HF}}^{2}}$ (Rivera et al. 2016b).

Differences occurring between maneuvers were assessed by ANOVA with the subsequent Sidak's multiple comparisons test. Statistical significance changes were defined as *P* < 0.05.

## Results

**Figure 2:**
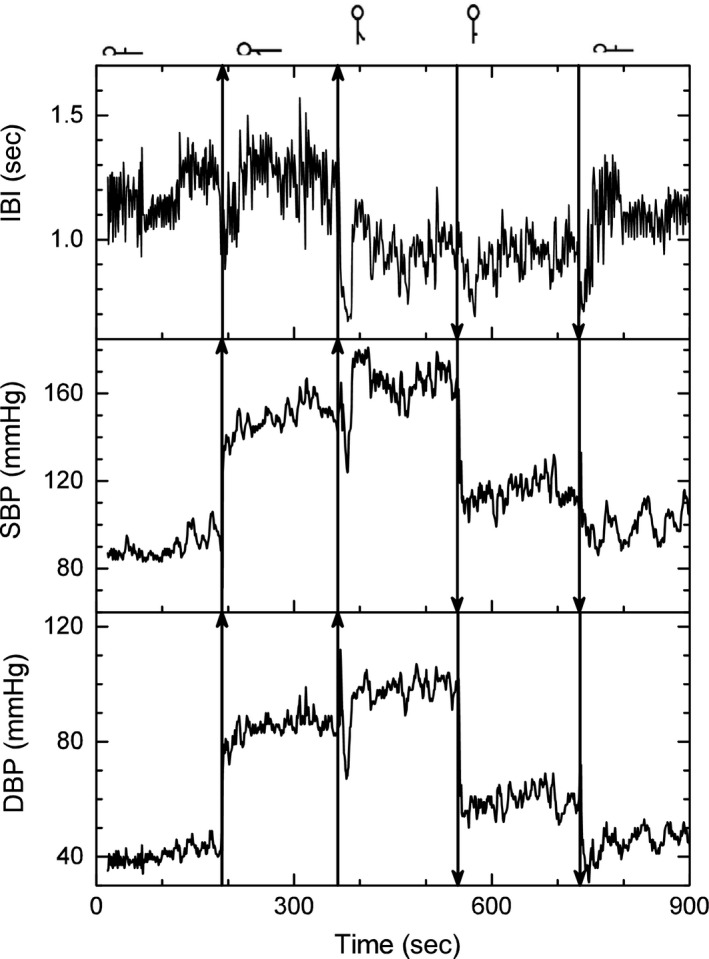
Temporal records from the Portapres^®^. Panels from top to bottom correspond to interbeat interval, systolic blood pressure, and diastolic blood pressure of a typical young subject during the five maneuvers: (1) supine position with the hand at heart level, (2) supine position with one arm dependent, (3) upright with the arm dependent, (4) upright with the arm at the heart level, and (5) supine with the arm at the heart level.

**Figure 3:**
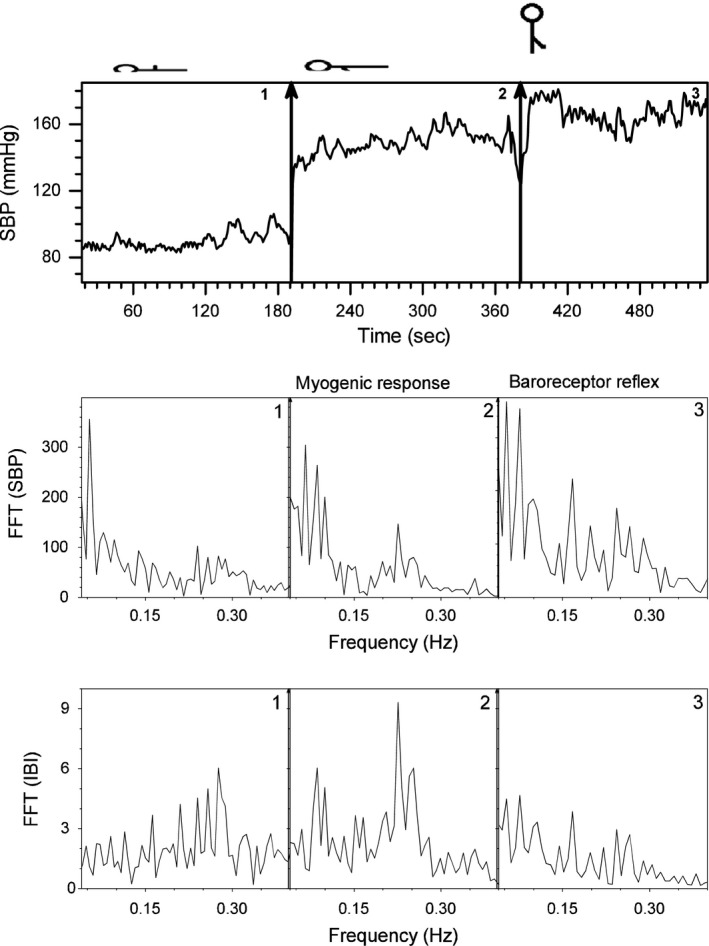
Schematic detail of Figure 2 (systolic blood pressure (SBP) (upper panel), Fourier Transform of SBP (middle panel), and of interbeat interval (lower panel)). The entrance of the myogenic activity, baroreceptor activity, and the Mayer waves (typical of sympathetic effects) are shown with arrows. On spectral analysis, the myogenic activity with the arm dependent was not associated with an increase in the amplitude of low frequency (LF), whereas the baroreceptor activity induced by standing was associated with a large peak at LF frequencies.

Typical records of the noninvasive short‐term time series measurements of the Portapres^®^ are shown in Figure 2 for the five maneuvers for a typical young healthy subject. It includes IBI, SBP, and DBP. Vertical lines mark the instant of position changes. A schematic detail of Figure 2 shows with arrows the entrance of the myogenic activity, baroreceptor activity, and the Mayer waves typical of sympathetic effects (Fig. 3). On spectral analysis, the myogenic activity with the arm dependent was not associated with an increase in the amplitude of LF, whereas the baroreceptor activity induced by standing up was associated with a large peak at LF frequencies.

**Figure 4:**
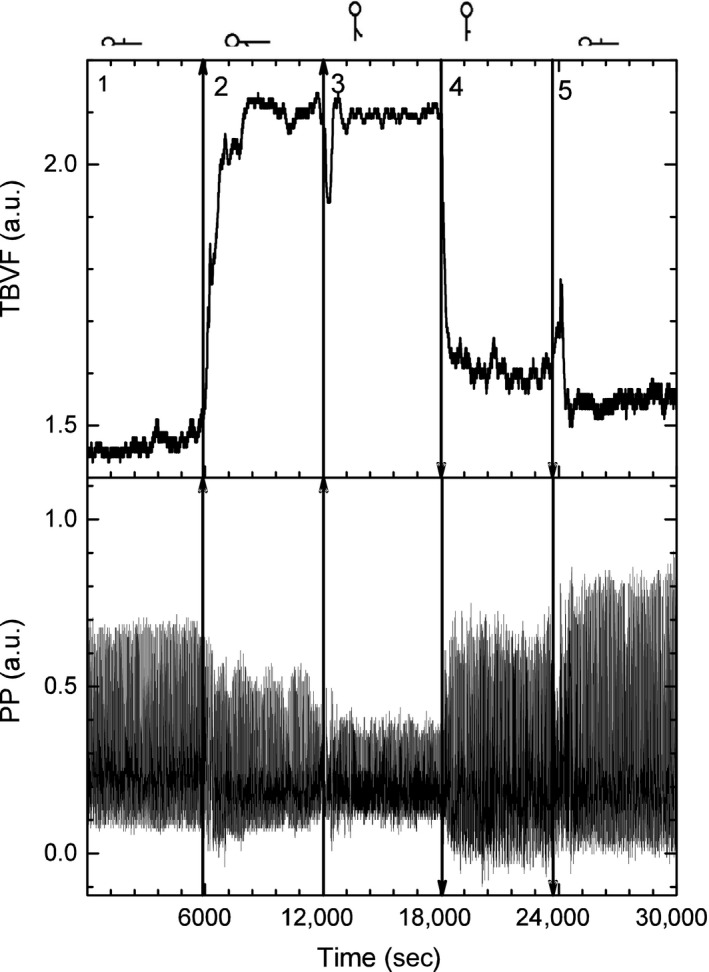
Noninvasive temporal records from the photopletysmograph of the pulsatile arterial skin blood flow pulse pressure, AC recording (lower panel), and the nonpulsatile total blood volume of the finger, DC recording (upper panel) of a typical young subject during the five maneuvers.

The photopletysmographic noninvasive temporal records of nonpulsatile TBVF and PP of a typical young subject during the five maneuvers are plotted in Figure 4.

**Figure 5:**
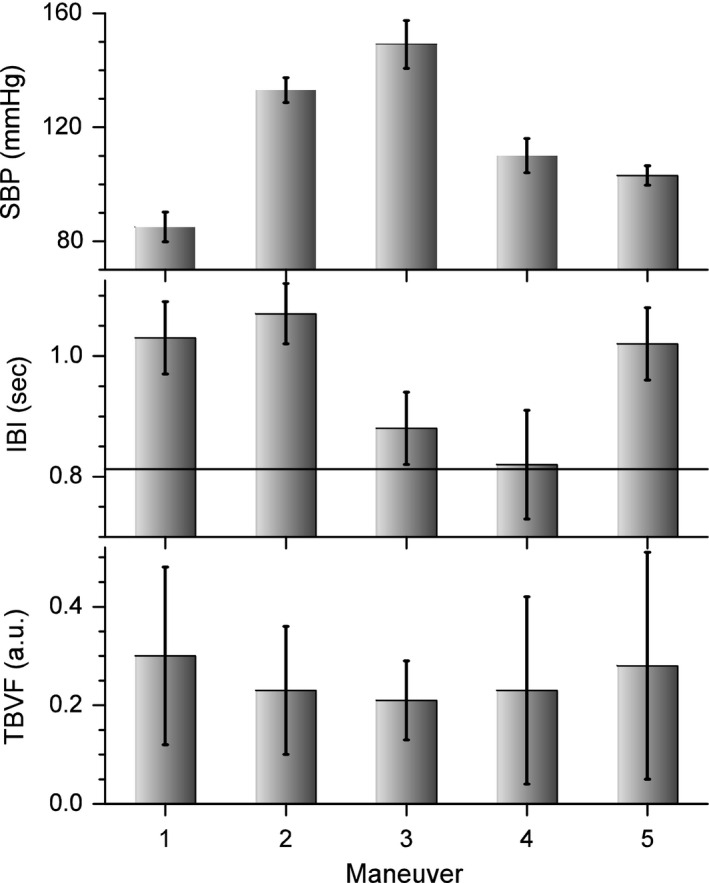
Mean and standard deviation of the interbeat interval (upper panel), systolic blood pressure (middle panel), and total blood volume of the finger (lower panel) of all the subjects during the five maneuvers: (1) supine position with the hand at heart level, (2) supine position with one arm dependent, (3) upright with the arm dependent, (4) upright with the arm at the heart level, and (5) supine with the arm at the heart level.

Numerical results for the parameters of HR variability for the different maneuvers are summarized in Table 1, for SBP data is reported in Table 2, and of TBVF in Table 3. Values corresponding to the mean (μ) ±standard deviation (SD) of the parameters from all the subjects are plotted in Figure 5 for IBI (up), SBP (middle), and TBVF (bottom).

**Table 1:** Interbeat intervals characteristic parameters

Maneuver	μ	SD	*Sk*	κ	*m*_IBI_	LF/HF	*r*_F_
Supine position with the hand at heart level (1)	1.03	0.06	−0.379	1.239	−0.08 ± 0.08	0.45	0.499
Supine position with one arm dependent (2)	1.07	0.05	−0.627	1.668	0.08 ± 0.09	0.40	0.314
Upright with the arm dependent (3)	0.88	0.06	−0.061	0.492	−0.8 ± 0.2	0.78	0.390
Upright with the hand at heart level (4)	0.82	0.09	−0.545	0.645	−0.3 ± 0.1	0.80	0.341
Supine position with the hand at heart level (5)	1.02	0.06	−0.702	1.468	−0.1 ± 0.2	0.49	0.403

Mean (μ), standard deviation (SD), skewness (*sk*), kurtosis (κ), power spectral density (PSD) slope (m_IBI_), low frequency (LF), high frequency (HF), $r_{f}=\sqrt{{\left(\text{LF}\right)}^{2}+{\left(\text{HF}\right)}^{2}}$.

**Table 2:** Systolic Blood Pressure characteristic parameters

Maneuver	μ	SD	*Sk*	*Κ*	*m*_SBP_	LF/HF	*r*_F_
Supine position with the hand at heart level (1)	85	5.2	−0.387	0.649	−0.8 ± 0.2	1.15	13.7
Supine position with one arm dependent (2)	133	4.4	0.186	0.175	−0.8 ± 0.2	1.31	8.3
Upright with the arm dependent (3)	149	8.4	−0.402	−0.597	−0.5 ± 0.1	1.16	26.1
Upright with the hand at heart level (4)	110	6.0	0.830	0.375	−0.9 ± 0.2	1.65	14.0
Supine position with the hand at heart level (5)	103	3.4	0.175	−0.105	−0.6 ± 0.2	1.17	9.7

Mean (μ), standard deviation (SD), skewness (*sk*), kurtosis (κ), power spectral density (PSD) slope (*m*_SBP_), low frequency (LF), high frequency (HF), $r_{f}=\sqrt{{\left(\text{LF}\right)}^{2}+{\left(\text{HF}\right)}^{2}}$.

**Table 3:** Nonpulsatile total blood volume of the finger characteristic parameters

Maneuver	μ	SD	*sk*	κ
Supine position with the hand at heart level (1)	0.30 ± 0.05	0.18 ± 0.09	0.752 ± 0.02	−0.634 ± 0.005
Supine position with one arm dependent (2)	0.23 ± 0.02	0.13 ± 0.08	0.880 ± 0.01	−0.118 ± 0.008
Upright with the arm dependent (3)	0.21 ± 0.05	0.08 ± 0.09	0.877 ± 0.02	0.468 ± 0.007
Upright with the hand at heart level (4)	0.23 ± 0.07	0.19 ± 0.08	0.756 ± 0.03	−0.541 ± 0.006
Supine position with the hand at heart level (5)	0.28 ± 0.05	0.23 ± 0.1	0.869 ± 0.01	−0.470 ± 0.009

### Condition 1: supine position with the hand at heart level

**Figure 6:**
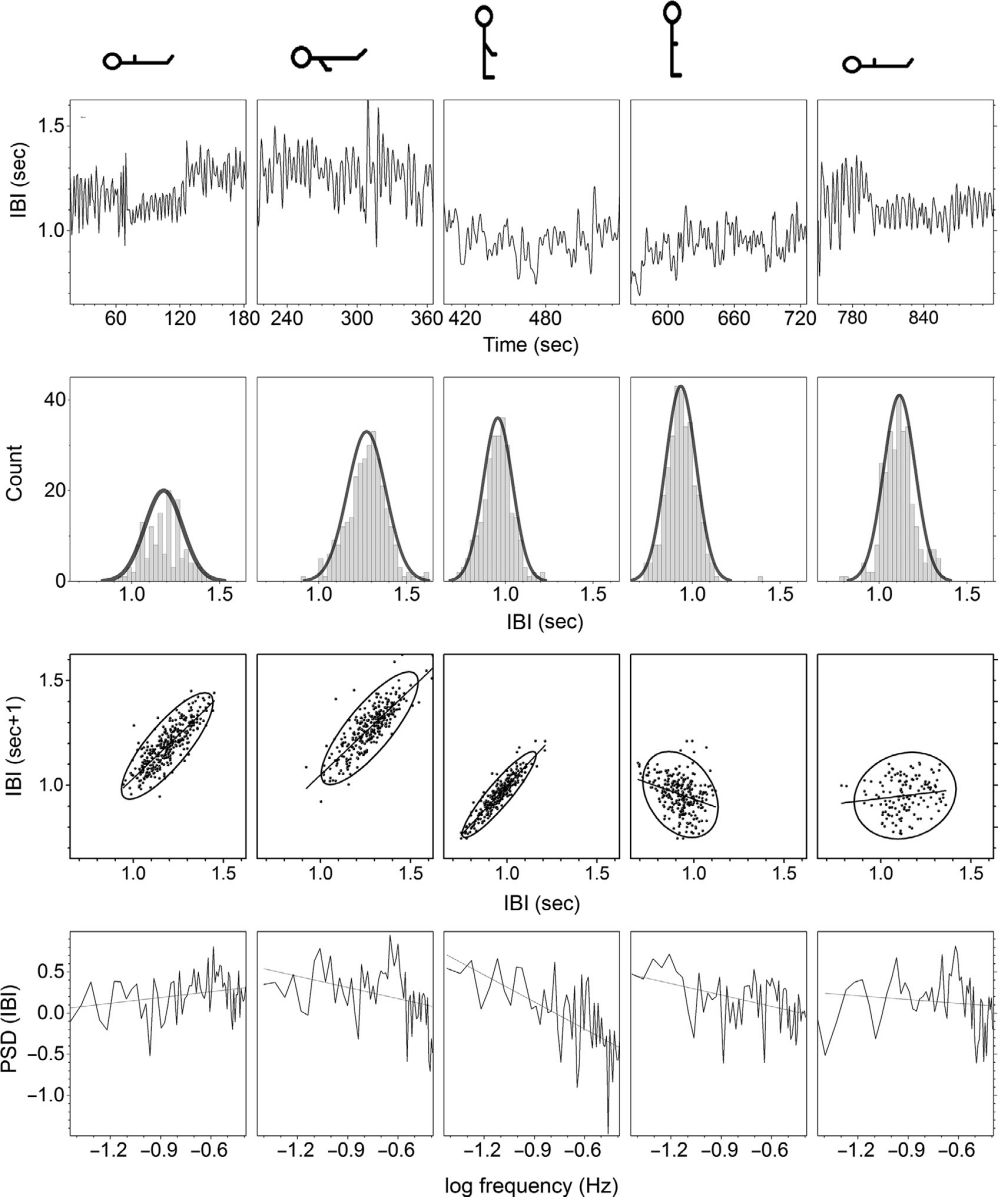
Interbeat interval signal of a typical young subject. Each column corresponds to the different position illustrated on the top. Panels from top to bottom illustrate the time series, histogram, Poincaré plot, and PSD.

**Figure 7:**
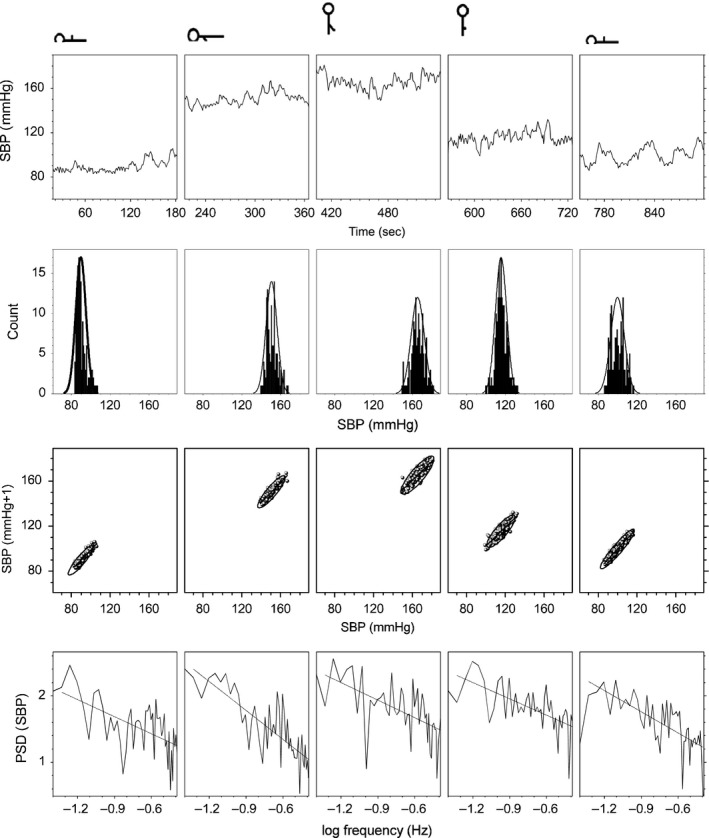
Systolic blood pressure signal of a typical young subject. Each column corresponds to the different position illustrated on the top. Panels from top to bottom illustrate the time series, histogram, Poincaré plot, and power spectral density.

This position is considered as the basal condition. In this condition, IBI for healthy humans is around one beat per second (Fig. 5 and Table 1), SBP 85 mmHg (Fig. 5 and Table 2), and DBP 50 mmHg. IBI has a random variation, with a PSD slope, not statistically different from zero at the *P* = 0.05 level (Fig. 6 and Table 1). SBP is scale invariant, with a PSD slope of 0.8 ± 0.2 (Fig. 7 and Table 2). There are no Mayer waves, oscillations at 0.1 Hz (Fig. 3). Basal value of the nonpulsatile TBVF is 0.3 ± 0.2 (Fig. 5 and Table 3), and PP is 1.46 ± 0.02.

### Condition 2: supine position with one arm dependent

**Figure 8:**
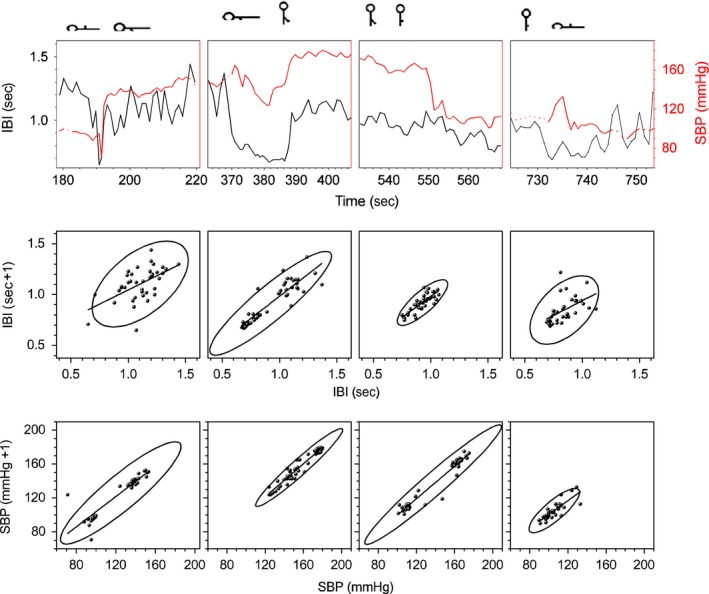
Signal on the transition regions of a typical young subject. Each column corresponds to the different position changes illustrated on the top. On the top row are the IBI (black) and systolic blood pressure (SBP) (red) time series, Poincaré plots of the interbeat interval is on the middle row, and Poincaré's of the SBP on the bottom row.

After the upper extremity was lowered, the nonpulsatile TBVF increased abruptly decreasing its variability and remained stable as long as the arm was kept below the heart level (Fig. 4 and Table 3). The pulsatile SBF decreased significantly (40 ± 10)% with respect to the basal level, remained low as long as the arm was kept below the heart level, and was inversely related to the increase in the blood volume of the finger. This reflects venous and capillary congestion and probably an increase in tisular pressure, whereas the lower amplitude of the pulsatile flow reveals vasoconstriction. IBI did not change during the supine position with a single upper limb dependent (Table 1). Furthermore, histogram, Poincaré plot, and spectral analysis of IBI are similar to basal conditions (Fig. 6). The PSD slope of IBI in the transition region at the *P* = 0.05 level, is not significantly different from zero, supporting the idea that the HR does not change in supine position when the arm was lowered (Fig. 6 and Table 1) making unlikely that the variation in BP parameters was due to an increase in cardiac output or baroreceptor activation. Supporting, in addition, the absence of the BRR, there are no Mayer waves (oscillations at 0.1 Hz of frequency) when the subject is supine with the arm dependent (Fig. 3). SBP and DBP increased significantly, almost 45 mmHg (50%) with respect to its basal values (Fig. 2). This change is proportional to the distance (*h*) of the lowered arm from the heart. Even when in supine position, the PSD slope of SBP at a *P* = 0.05 level, statistically is not significantly different from the basal conditions (Fig. 7 and Table 2), in the transition region SBP has a growing tendency (slope statiscally significant different of 0 at the *P* = 0.05 level). The pulse pressure widened from 40 ± 3 to 70 ± 4 mmHg (Fig. 4). The rise in all these parameters persisted during the entire time the arm was kept down. In the transition region of supine position with the hand at heart level to the lowered arm, Poincaré plots (Fig. 8) show a random HR variability, and a strong correlation with the values of SBP.

### Condition 3: Upright with the arm dependent

In upright position, with a dependent limb, there was a further increase of about 20% compared to the basal position, in the variables indicating vasoconstriction (PR, amplitude of the pulsatile SBF, and increased PP), and in all the BP variables, in particular SBP increases 60 mmHg with respect to basal values. SBP becomes less correlated, as can be seen in the PSD (Fig. 7, and Table 2). Thus, there is not a global pressure modification. Both BP and HR show Mayer waves, oscillations at 0.1 Hz (Figs. 3, 6 and 7), indicating the entrance of the BRR. IBI decreased 15%, and present a statistical significant change of the PSD slope from zero, becoming a 1/f scale‐invariant distribution (Fig. 6, and Table 1). In the transition region, from supine to upright positions with the dependent arm, Poincaré plots (Fig. 8) showed also the entrance of BRR because IBI signal become more autocorrelated. Moreover, when the subject stands up, there are reciprocal changes of SBP and HR, also a characteristic of the BRR (Fig. 8). TVBF increased slightly (Table 3), about 10% from the previous value, presenting a smaller variance and a clear notch (Fig. 4), in the nonpulsatile venous volume wave, that indicates the entrance of the BRR at the venous side. When the subject assumes the upright position with his arm down, there is a further decrease in the amplitude of the pulsatile arterial skin blood flow given by the activation of the baroreceptor reflex and neural vasoconstriction. The venous volume does not change although there is transient decrease in venous volume probably induced by venous constriction of the baroreceptor activity.

### Condition 4: upright with the arm at the heart level

All the variables indicating vasoconstriction (amplitude of SBF, PR, PP) returned in 3–4 beats to the previous value (before lowering the arm) and the TBVF also returned to its previous value of basal oscillation. When the subject is upright with his arm at the heart level, the amplitude of the pulsatile skin blood flow immediately increases, whereas the nonpulsatile blood volume decreases (Fig. 4). The withdrawal of the myogenic response produces vasodilatation and gravity decreases the venous congestion. SBP and DBP decreased rapidly up to 50 mmHg (Fig. 1), going to values closer to basal conditions. HR was maintained at the previous maneuver as seen on panels 3 and 4 of the IBI time series, Poincaré plot, and spectral analysis (Fig. 6). In the transition region, SBP has a similar Poincaré plot as in the transition from supine position with hand at the heart level to the arm lowered (Fig. 8). This suggests that withdrawal reflex is taking place.

### Condition 5: supine with the arm at the heart level

When reassuming the supine position with the hand at the heart level, the pulsatile skin blood flow has some rebound increase in amplitude and the venous volume return to basal values making the return of all the variables to their basal values, including those reflecting vasoconstriction and those reflecting changes of BP and HR.

## Discussion

These experiments were designed to explore the possibility of separating the gravity‐induced myogenic vascular vasoconstriction response from the neural vasoconstriction generated by the BRR during active standing. We recorded, by lowering the arm in the supine position (second maneuver), the isolated regional changes of BP and the SBF induced by the gravity stress in the dependent arm. Assuming the upright position with the arm down (third maneuver), we analyzed the generalized vasoconstrictive changes of BP and SBF induced by the BRR superimposed to the gravity‐induced myogenic vasoconstriction, as the arm was kept lowered during the upright position. In all subjects, lowering the arm in the supine position induced a vasoconstriction response with an increase in SBP, DBP, and an important rise of regional peripheral resistances, and an increase of the PP. We also demonstrated that there was a decrease in the amplitude of the arterial pulsatile SBF due to vasoconstriction and an increase in the total volume of blood in the cutaneous vessels of the finger. In the second position, all these changes were regional as the BRR was not activated. This is suggested by the fact that the subjects remained in the supine position, and the changes of BP were not associated with reciprocal changes of the HR. The central command was not activated because the arm was lowered passively and the HR did not increase. Therefore, vasoconstriction was likely to be due to a local myogenic response induced by the pressure of gravity. The facts that militate for a myogenic response induced by gravity are:

1. the increase of BP was directly proportional to the distance *h* of the dependent arm to the heart level considered as the hydrostatic neutral point;
2. the increase of BP was not associated with changes of HR and therefore not likely to be secondary to BRR activation or central command;
3. there were no oscillations of BP at 0.1 Hz (characteristic of BRR activation). The peripheral resistance increased, the amplitude of the pulsatile SBF decreased, and the PP widened, indicating vasoconstriction. It was unlikely that an adrenergic stimulation could induce the arterial vasoconstriction because intensive study of this response has shown that it persists in the lower extremities with epidural blockade (Henriksen et al. 1983; Henriksen 1991), and in the upper extremities with axillary blockade (Vissing et al. 1987). It is present in patients with denervated skin flaps (Zoltie et al. 1989), in paraplegics and tetraplegic patients (Kooigman and de Hoog 2007), and because there is vasodilatation in space in a microgravity situation (Norsk et al. 2006). Several careful pharmacological studies have demonstrated that arterial vasoconstriction in a dependent arm is not secondary to alpha adrenergic activation (Crandall et al. 2002; Okasaki et al. 2005).

When the subject is upright with the arm dependent, there is a further increment of BP that is related to BRR activation. This response is superimposed to the already existing gravity‐induced myogenic vasoconstriction. The facts that support that there is BRR activation and sympathetic vasoconstriction when standing with the arm dependent are:

1. there is a further increase of BP that occurs when the subject stands up. It is known that in assuming the upright position, there is a generalized redistribution of the blood volume below the heart and a sudden fall of BP followed by BRR activation and generalized vasoconstriction (Berne 1981; Rowell 1993; Berne and Levy 2001; Wieling and van Lieshout 2008),
2. there are oscillations at 0.1 Hz that are characteristic of BRR activation, the BP fluctuates in a characteristic fashion on visual inspection (Mayer waves), and there is more variability of BP typical of reflex control, and
3. the HR increases in a reciprocal way to the increment of BP. There was an additional increase in peripheral resistances and a decrease in the amplitude of the pulsatile SBF indicating an increment of vasoconstriction.

All these changes were readily observed in our subjects. This is also supported by the last phase of the experiment: when the subject, while still standing, elevates his arm to the heart level, the BP in the arm abruptly descends due to the withdrawal of the gravity‐induced myogenic local response, whereas the BRR maintains the BP slightly above the basal level. The HR continues to increase with respect to its basal levels and the low‐frequency oscillations at 0.1 Hz persists. The regional peripheral resistance decreases and the amplitude of the SBF increases, demonstrating a reduction in arterial and arteriolar vasoconstriction. Lastly, when the subject lies down, with his arm at the heart level, the BP, HR, peripheral resistances return to basal values, as the BRR is not active and there is no gravity stress on the arm and finger at the heart level. The increase in venous pressure, arterial vasoconstriction, and in BP seems to be due, at least in part, to the action of gravity, as both, venous and arterial pressure are increased by gravity. The venous pressure at the feet has been shown to increase from 5 mmHg in supine position to 100 mmHg (13.33 kPa) in the upright position, whereas the SBP increases from 120 mmHg (15.99 kPa) in the supine position to 200 to 220 mmHg (26.66 to 29.33 kPa) in the upright position. Meanwhile, supine BP is approximately the same at the heart level, the head, and the feet because the gravitational vector does not act upon the arterial and venous system either below or above the heart (Berne 1981; Kane and Sternheim 1988; Rowell 1993; Berne and Levy 2001).

The important finding of our experiment is the demonstration that it is possible to separate or segregate the myogenic vascular response (MVR) induced by gravity and the vascular response induced by the BRR, in vivo*,* in healthy young humans. MVR was discovered by Bayliss 1902 by clamping and uncampling the aorta for several seconds in the experimental animal (Bayliss 1902). Fifty years later, in 1952, Bjorn Folkow, demonstrated the MVR by increasing and decreasing at various pressures the arterial intraluminal pressure of isolated denervated arteries (Folkow 1952). Since that time, the myogenic response has been intensively studied mostly in isolated segments of arteries and arterioles (Davis and Hill 1999) but it has proven to be recalcitrant to be studied in vivo in experimental animals and in humans. Some few researchers have observed the VMR (Richardson and Shepherd 1989), and it has been studied mostly in laboratories in isolated segments of arteries (see Davis and Hill 1999). The basic mechanism of myogenic vasoconstriction has been extensively studied in vitro. There are a variety of calcium‐mediated mechanisms that increase vascular smooth muscle cells contractility (Davis and Hill 1999; Brozovich et al. 2016).

Limitation of the study: Although the generalized BRR was assessed by HR variability, we also used the changes on the SBP and the spectral analysis that relates the sympathetic activity with baroreflex activation at 0.1 Hz (Mayer waves). One limitation of this study is that, as a noninvasive approach was taken, we could not record the muscle sympathetic nerve activity. This can be done in a future study, in order to characterize with some other method, the entrance of the BRR.

## Conclusion

We are bipeds, with our heads above our heart that have to live two‐thirds of our lives with our circulatory system counteracting the action of gravity. In fact, it is likely that the BP, in microgravity conditions, is the same in all regions of the body, no matter the position of the body, as the BP under this condition, is a function only of the cardiac output as all the vessels are vasodilated (Norsk et al. 2006). The BRR is an extraordinary mechanism that is aided by the gravity‐induced MVR to keep the BP and the blood flow autoregulated in the upright position.

The vascular myogenic response is thought to be present in the kidney, the brain, and the coronary arteries (Davis and Hill 1999). Our findings reveal that the gravity‐induced myogenic vascular response can be studied in vivo in humans and its response can be separated from the vascular response induced by baroreceptor activation using a relatively simple and straightforward maneuver. This method to study the myogenic response of the vasculature may be useful in the future for several important diseases, including hypertension and orthostatic hypotension.

## Conflict of Interest

The authors have no conflict of interest to report.
